# Cross-Disease Transcriptomic Signatures Shared by NAFLD and MI: Multicohort Machine-Learning Analysis and Exploratory IDI1 Expression Assessment

**DOI:** 10.3390/ijms27209390

**Published:** 2026-10-21

**Authors:** Jilei Qiao, Shuran Chen, Shuo Li, Wenbo Shao, Yiran Zhang, Ying Huang, Yangjing Xie

**Affiliations:** 1 Department of Cardiology, First Affiliated Hospital of Anhui Medical University, Hefei 230032, China; 2014010060@stu.ahmu.edu.cn (J.Q.); 2645011672@stu.ahmu.edu.cn (S.L.); 2645011442@stu.ahmu.edu.cn (W.S.); 2 Department of General Surgery, First Affiliated Hospital of Anhui Medical University, Hefei 230032, China; chenshuran668@gmail.com; 3 The First Clinical College, Anhui Medical University, Hefei 230022, China; 2313010118@stu.ahmu.edu.cn

**Keywords:** non-alcoholic fatty liver disease, myocardial infarction, IDI1, machine learning, liver–heart axis

## Abstract

The molecular features shared by non-alcoholic fatty liver disease (NAFLD) and myocardial infarction (MI) remain incompletely understood. We analyzed six microarray cohorts from the GEO database to identify shared disease-associated transcriptomic features across independent NAFLD and MI populations. Weighted gene co-expression network analysis, differential expression analysis, and fuzzy c-means clustering across ordered cross-sectional NAFLD groups were used to define candidate genes. LASSO regression, random forest, and SVM-RFE identified consensus computational candidates, which were examined using exploratory classification, receiver operating characteristic curves, calibration curves, decision curve analysis, and SHapley Additive exPlanations. *IDI1* was selected for exploratory expression assessment by qPCR in patients with established MI and in a mouse model of acute ischemic myocardial injury. Fifty-three shared candidate genes were identified, and five genes were retained by all three feature-selection methods. Single-gene ROC analyses showed *SULF2*, *GADD45A*, *LIN7A*, and *IDI1* within the analyzed case-control datasets. *IDI1* expression was higher in patients with MI than in healthy controls (*p* = 0.004), as well as in mice blood (*p* = 0.034) and infarcted myocardium (*p* = 0.0072) following LAD ligation. These findings indicate shared transcriptomic links between NAFLD and MI, supporting an association between *IDI1* and myocardial injury.

## 1. Introduction

Non-alcoholic fatty liver disease (NAFLD), recently redefined as metabolic dysfunction-associated steatotic liver disease (MASLD), affects approximately one-quarter of the global adult population [1,2,3,4]. Cardiovascular disease, rather than liver-related complications, is the leading cause of death among patients with NAFLD, and myocardial infarction (MI) contributes substantially to this burden [5,6,7].

NAFLD and MI share metabolic risk factors, with insulin resistance contributing to both hepatic lipid accumulation and atherosclerosis [8,9]. This pathophysiological connection is often described as the liver–heart axis. Clinical indices such as the fatty liver index (FLI) and fibrosis-4 index (FIB-4) have therefore been proposed as supplements to conventional cardiovascular risk scores [10,11]. However, treating NAFLD as a broad clinical exposure may obscure the heterogeneous molecular pathways that contribute to myocardial injury and may limit the identification of actionable therapeutic targets [12].

Recent studies have begun to investigate the molecular links between NAFLD and MI. In particular, Huang et al. [13] applied bioinformatic and machine-learning strategies to identify genes and molecular features shared between NAFLD and MI, providing evidence that the two diseases may converge on common transcriptional pathways. However, most bioinformatics studies have relied on single datasets, conventional correlation analyses, or limited experimental validation [14,15,16]. Further cross-cohort investigation is therefore needed to characterize shared disease-associated transcriptomic signals while distinguishing such associations from prospective risk prediction or causal mechanisms.

Recent clinical and experimental insights emphasize that the liver–spleen axis and age-related vascular changes have been discussed as potential contributors to the systemic inflammatory and vascular abnormalities associated with NAFLD. Previous studies have also reported an association between NAFLD and increased carotid intima–media thickness (cIMT), a surrogate marker of subclinical atherosclerosis. These observations provide a clinical context for investigating shared molecular features between NAFLD and MI; however, they do not establish a direct or causal pathway from NAFLD through cIMT progression to MI.

In this multicohort, cross-disease study, we combined weighted gene co-expression network analysis (WGCNA), differential expression analysis, and ordered cross-sectional expression pattern clustering to identify shared molecular signatures. Consensus feature selection using three machine learning algorithms (LASSO, random forest, and SVM-RFE) yielded a five-gene signature, which was evaluated in eight classification models. The leading biomarker was then assessed in peripheral blood from patients with MI and in a mouse MI model to provide experimental support for the transcriptomic findings. Because the NAFLD and MI datasets comprised different participants and no prospective NAFLD cohort with incident-MI follow-up was available, the study was not designed to predict future MI in patients with NAFLD.

## 2. Results

### 2.1. Identification of NAFLD-Related Co-Expression Modules Across Independent Cohorts

**Figure 1:**
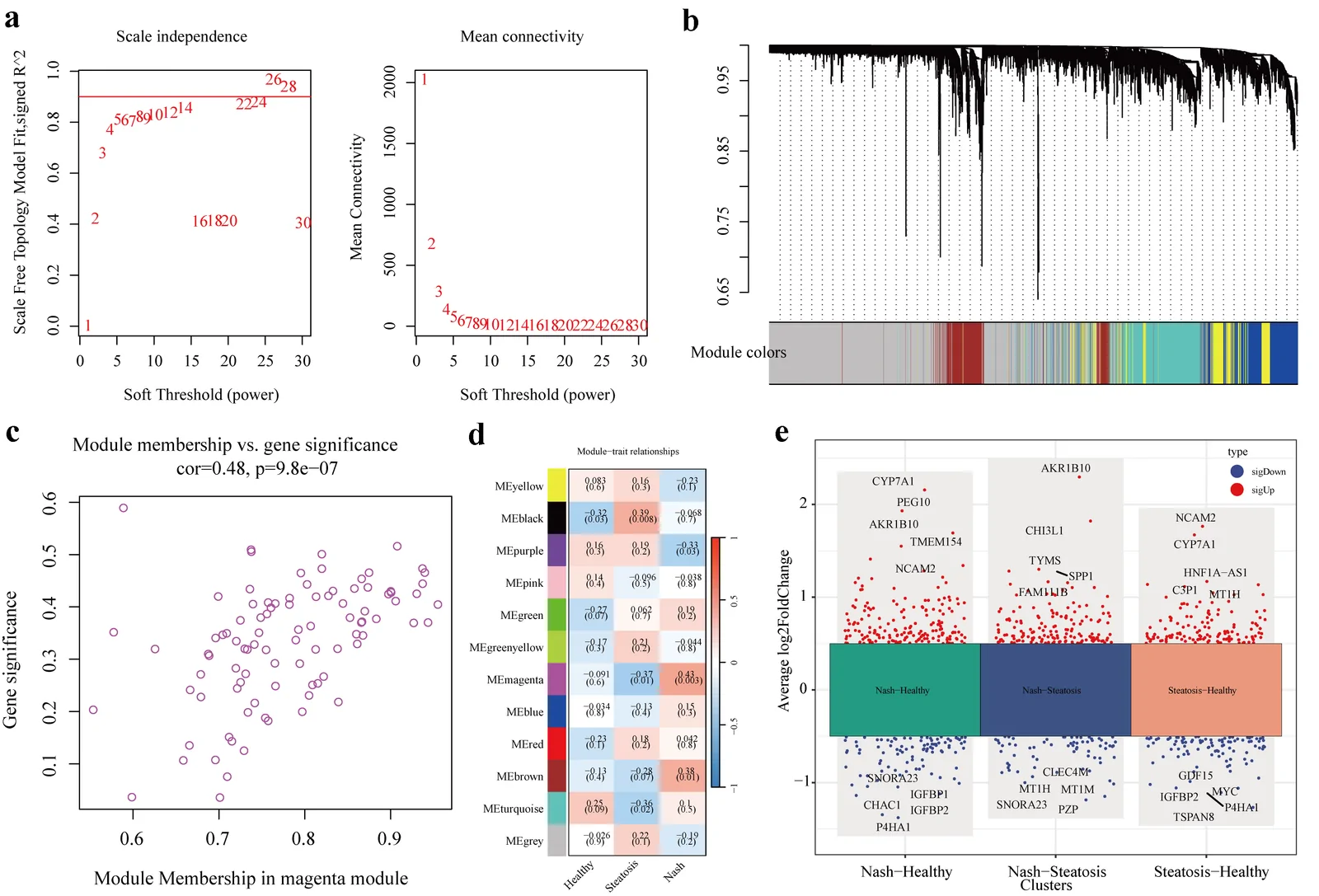
Identification of NAFLD-related co-expression modules in the GSE48452 cohort. (**a**) Selection of the soft-thresholding power (β = 14). The left and right panels show the scale-free topology fit index (signed R^2^) and mean connectivity, respectively, across candidate soft-thresholding powers. (**b**) Hierarchical clustering dendrogram of genes based on topological overlap. The colored bar below the dendrogram indicates module assignment; gray denotes unassigned genes. (**c**) Scatterplot showing the positive correlation between module membership (MM; x-axis) and gene significance for NASH (GS; y-axis) within the magenta module. (**d**) Heatmap of module-trait relationships across Healthy, Steatosis, and NASH samples. The blue-white-red color scale represents Pearson correlation coefficients from −1 to 1, with *p* values shown in parentheses. (**e**) Volcano plots of differentially expressed genes (DEGs) for NASH versus Healthy, NASH versus Steatosis, and Steatosis versus Healthy. Red and blue points indicate significantly upregulated and downregulated genes, respectively (|log_2_FC| >1 and adjusted *p* < 0.05).

WGCNA of the GSE48452 cohort identified 11 co-expression modules at an optimal soft-thresholding power of β = 14 (Figure 1a,b). The magenta module showed the strongest positive correlation with NASH (r = 0.43, *p* = 0.003), whereas the black module was significantly associated with Steatosis (r = 0.39, *p* = 0.008) (Figure 1d). Within the NASH-associated magenta module, module membership correlated with gene significance (r = 0.48, *p* < 0.001) (Figure 1c). Differentially expressed genes (DEGs) across disease stages were also identified in GSE48452 to characterize transcriptome-wide changes (Figure 1e). A parallel analysis of the independent GSE89632 cohort identified co-expression modules associated with Steatosis (r = 0.39, *p* = 0.002) and NASH (r = 0.39, *p* = 0.002). DEG analysis was also performed in GSE89632 to expand the candidate pool for subsequent cross-disease intersection.

### 2.2. Cross-Sectional Expression Patterns Across Ordered NAFLD Groups

**Figure 2:**
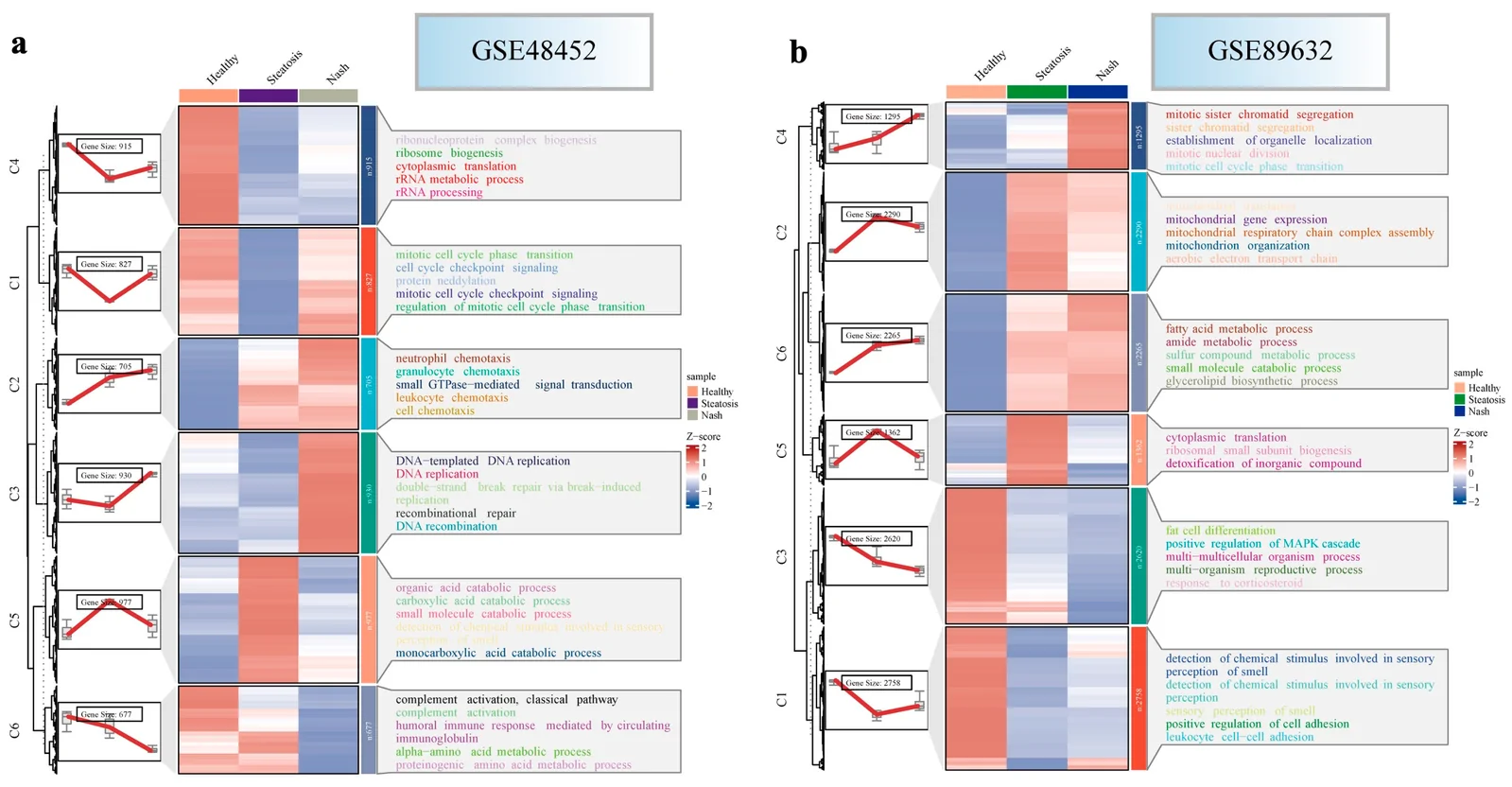
Mfuzz clustering and functional enrichment across two NAFLD cohorts. (**a**) Expression patterns of clusters C1–C6 and representative enriched Gene ontology (GO) terms across Healthy, Steatosis, and NASH samples in GSE48452; (**b**) Corresponding expression patterns and representative enriched GO terms for clusters in GSE89632.

Fuzzy c-means clustering of DEGs and genes from key WGCNA modules across the ordered healthy, steatosis, and NASH stages identified six cross-sectional expression clusters in each cohort. In GSE48452, clusters C2 and C3 showed higher mean expression across the ordered groups. C2 was enriched for neutrophil chemotaxis and inflammatory signaling, whereas C3 was enriched for DNA replication and damage-repair pathways (Figure 2a). In GSE89632, C2 peaked at the Steatosis stage and was enriched for mitochondrial gene expression and respiratory-chain assembly. C6 remained elevated in NASH and was primarily enriched for fatty acid metabolism (Figure 2b).

### 2.3. Identification of Shared Gene Signatures Between Nafld and Mi

**Figure 3:**
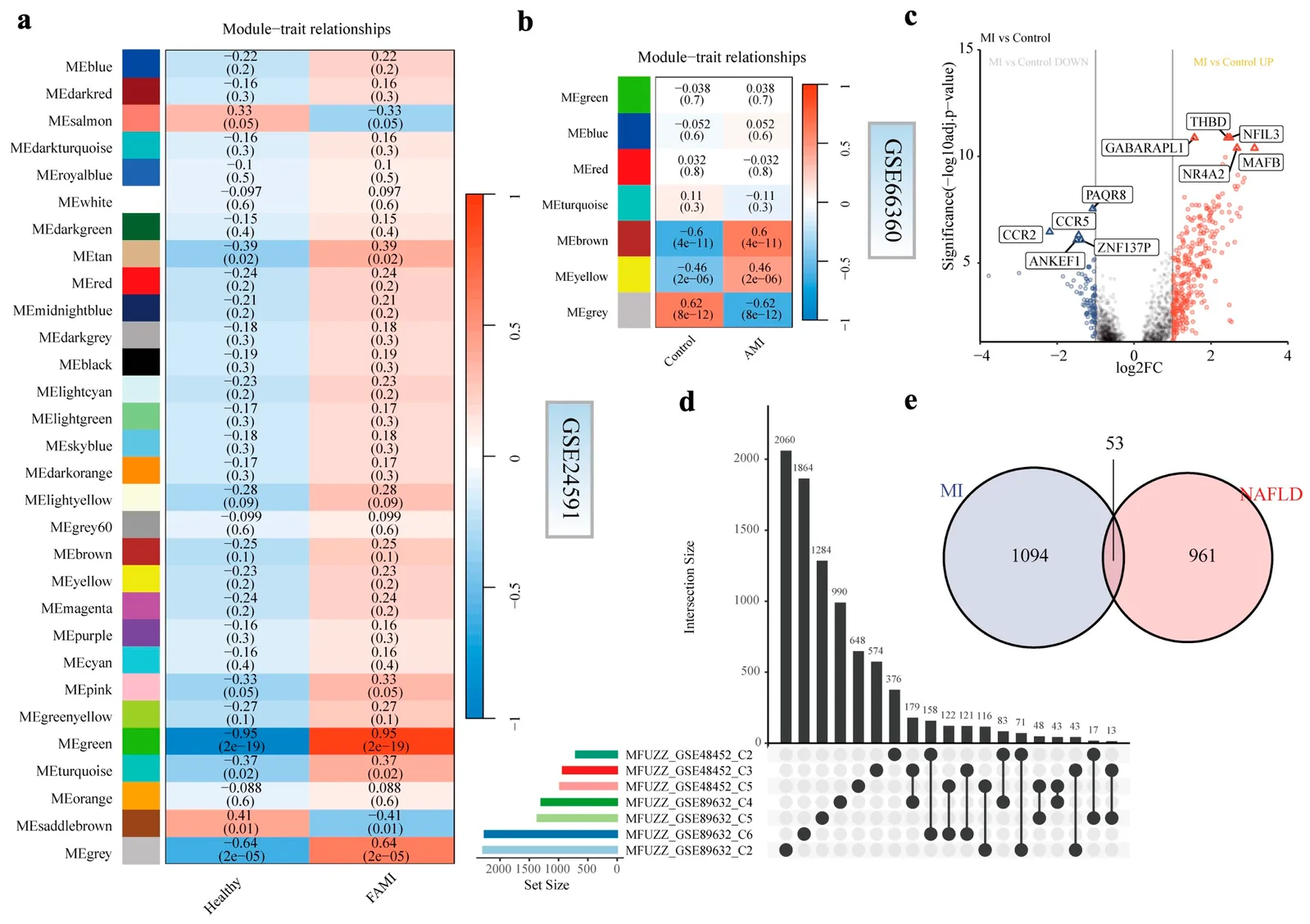
Identification of shared candidate genes between NAFLD and MI. (**a**,**b**) Heatmaps showing module-trait relationships in the GSE66360 and GSE24591 MI cohorts, respectively; (**c**) Volcano plot showing differentially expressed genes (DEGs) between MI and control samples in GSE66360; (**d**) integration of NAFLD-related candidate gene sets identified across the two NAFLD cohorts; (**e**) Venn diagram showing the intersection of the MI- and NAFLD-related gene sets, which yielded 53 shared candidate genes.

To identify shared molecular signatures, MI-associated co-expression modules were identified in GSE66360 and GSE24591 (Figure 3a,b), and DEGs between MI and control samples were identified in GSE66360 (Figure 3c). These analyses formed the MI candidate gene pool and established that GSE66360 contributed to upstream feature discovery. A NAFLD candidate gene set was derived by gene-level integration of selected expression clusters identified independently in the two NAFLD cohorts (Figure 3d). Intersecting the MI and NAFLD candidate sets yielded 53 shared genes (Figure 3e).

### 2.4. Consensus Computational Feature Selection

**Figure 4:**
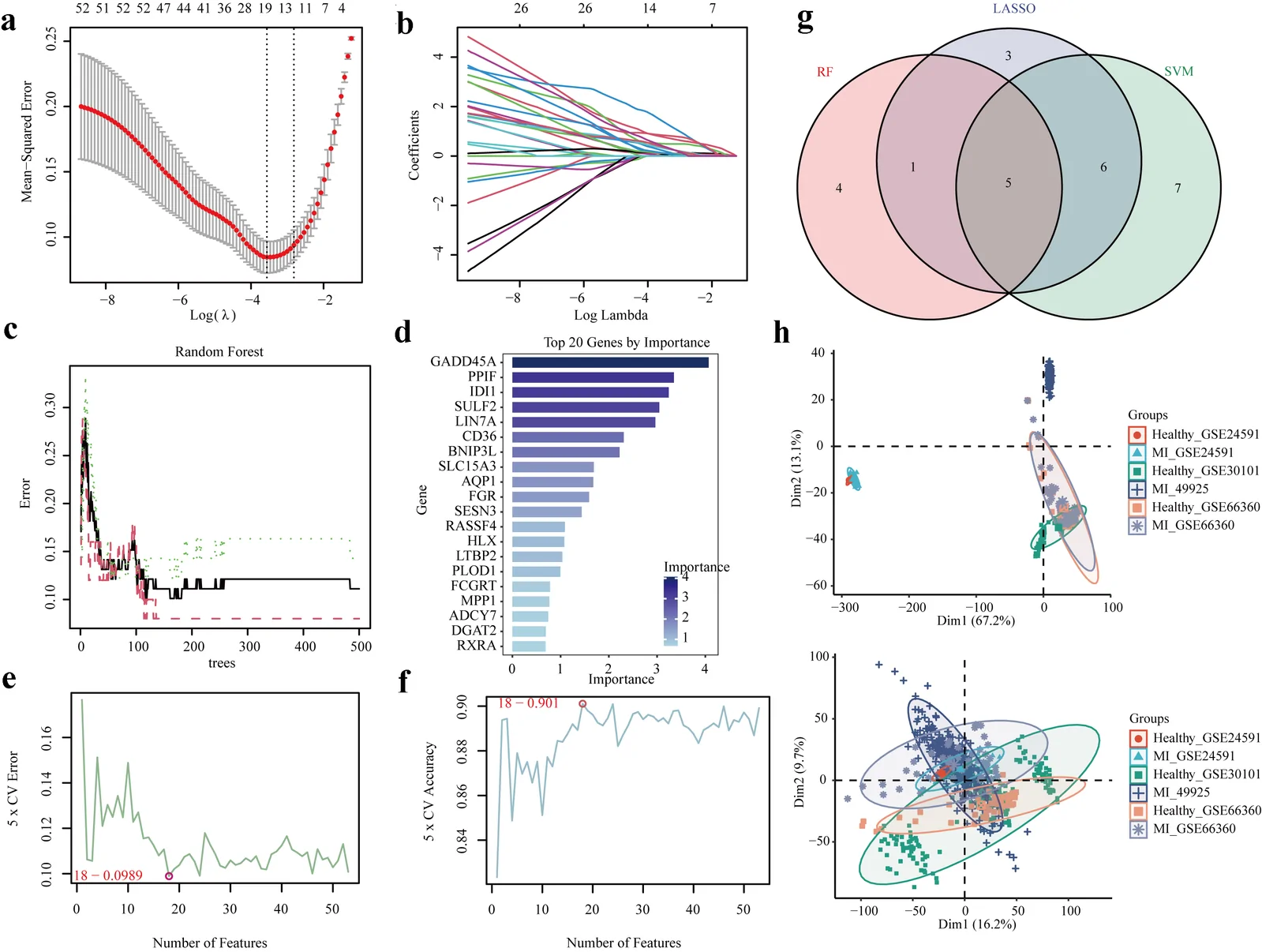
Machine learning-based feature selection and multicohort data integration. (**a**,**b**) Cross-validation error curve and coefficient-profile plot for LASSO regression; (**c**,**d**) relationship between out-of-bag error and the number of decision trees, and the top 20 candidate genes ranked by predictive importance in the random forest model; (**e**,**f**) cross-validation error and accuracy during SVM-RFE; (**g**) Venn diagram showing the intersection of candidate genes identified by LASSO, random forest, and SVM-RFE, which yielded five consensus genes; (**h**) principal component analysis (PCA) showing sample distributions for GSE24591, GSE30101, GSE49925, and GSE66360. The model-development pool comprised only the batch-corrected GSE24591, GSE30101, and GSE49925 datasets; GSE66360 is shown for comparison and was subsequently used as the secondary evaluation cohort.

Because the upstream 53 shared candidate genes were derived exclusively from GSE48452, GSE89632, GSE66360, and GSE24591 (independently of GSE49925), three machine learning algorithms were subsequently applied to prioritize consensus computational candidates. LASSO regression retained 15 genes with nonzero coefficients (Figure 4a,b). Random forest (RF) analysis ranked candidates by model importance and identified *GADD45A*, *PPIF*, and *IDI1* as the leading contributors (Figure 4c,d). SVM-RFE progressively eliminated features to retain a core set of MI-associated genes (Figure 4e,f). The intersection of the three methods yielded five consensus computational candidates: SULF2, GADD45A, LIN7A, IDI1, and AQP1 (Figure 4g). Before model construction, GSE24591, GSE30101, and GSE49925 were integrated and corrected for cross-cohort batch effects using ComBat. PCA was used to inspect sample distributions, with GSE66360 displayed alongside the three model-development datasets for comparison (Figure 4h). Only the corrected data from GSE24591, GSE30101, and GSE49925 were stratified by outcome and randomly divided into a training set (70%) and an internal test set (30%). GSE49925 contains mixed coronary phenotypes, but the original integrated workflow used a dataset-level outcome label; consequently, the integrated classifier cannot be interpreted as estimating acute-MI diagnostic accuracy. GSE66360 was excluded from model fitting and internal data partitioning and was subsequently evaluated as a secondary evaluation cohort. Because GSE66360 had contributed to upstream candidate-gene discovery, this evaluation was not considered fully independent external validation.

### 2.5. Exploratory Classification and Secondary Evaluation

**Figure 5:**
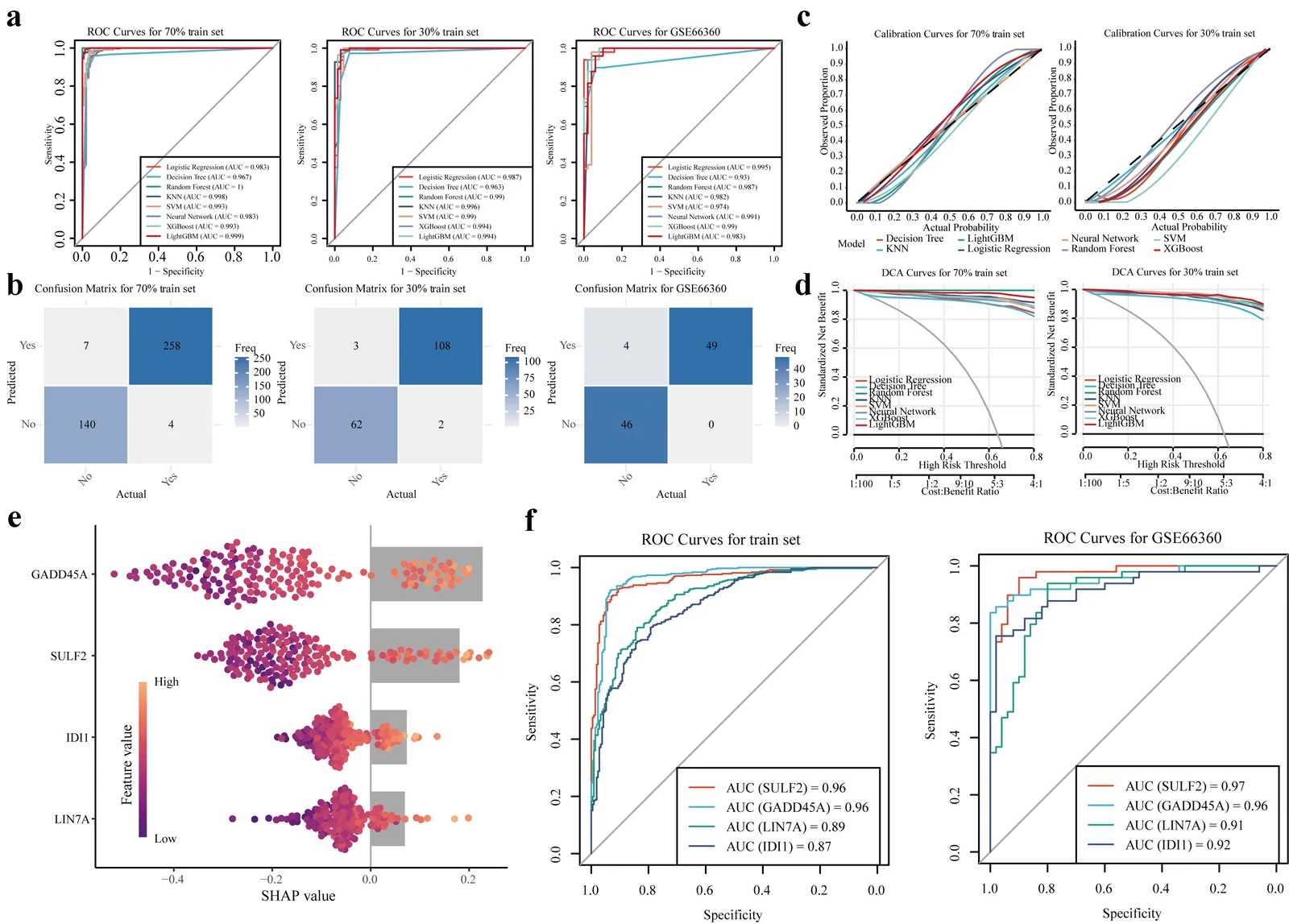
Performance and interpretability of the machine learning models. (**a**) ROC curves for the eight models in the training set, internal test set, and GSE66360 secondary evaluation cohort; (**b**) confusion matrices showing the classification performance of the KNN model; (**c**) calibration curves for the predictive models; the diagonal line indicates perfect calibration; (**d**) DCA showing model net benefit across threshold probabilities; (**e**) SHAP summary plot showing the importance and individual contributions of the consensus genes in the KNN model; (**f**) single-gene ROC curves for the four leading contributors in the training and secondary evaluation cohorts.

GSE49925 could not be disaggregated into individual-level acute-MI labels in public GEO metadata; the training and internal test set metrics reflect only dataset-level separation between heterogeneous coronary samples and healthy controls and cannot be used as evidence of MI discrimination; only the secondary evaluation in GSE66360 (49 confirmed AMI vs. 50 healthy controls) specifically reflects acute-MI case-control separation (Figure 5a,b). Calibration curves and decision curve analysis were exploratory and do not establish accurate clinical risk estimation, a clinically actionable threshold, or patient benefit (Figure 5c,d). SHAP values describe model-level feature contributions only and do not imply biological causality (Figure 5e). Single-gene ROC curves describe case-control discrimination within the analyzed data and do not demonstrate future risk prediction (Figure 5f).

### 2.6. Exploratory Assessment of Idi1 Expression in Human and Mouse MI Samples

**Figure 6:**
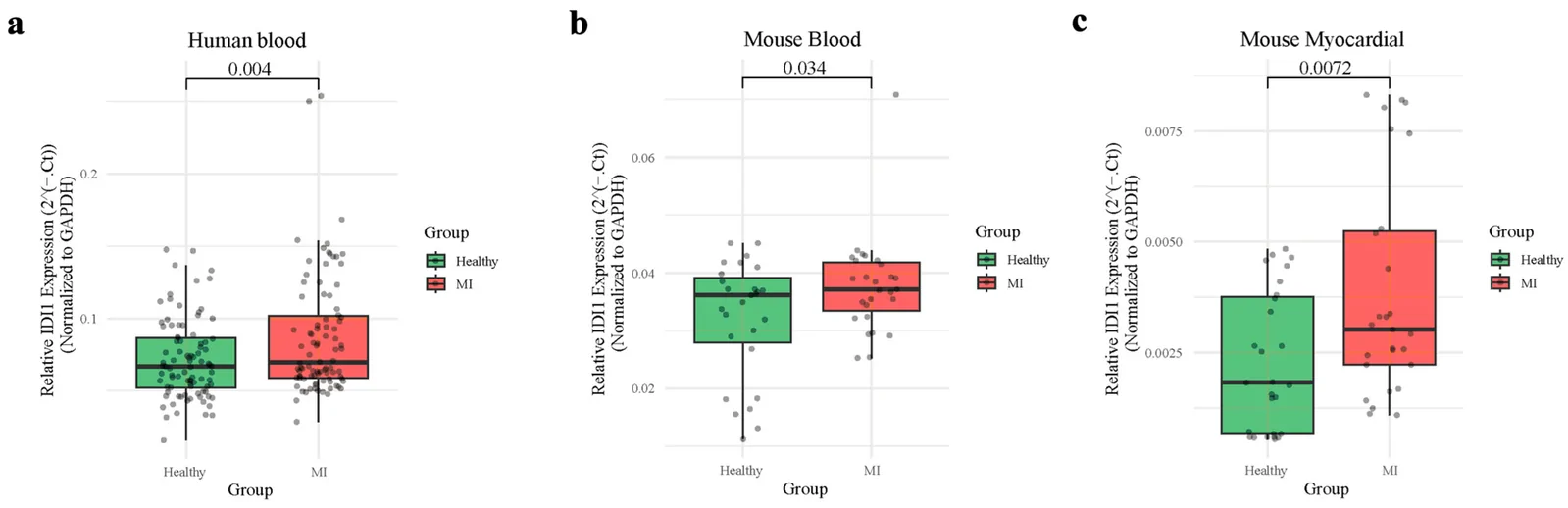
Exploratory assessment of IDI1 expression in human samples and a mouse MI model. (**a**) Relative *IDI1* mRNA expression in peripheral blood from patients with MI and healthy controls (*p* = 0.004). (**b**,**c**) Relative *Idi1* mRNA expression in peripheral blood (b; *p* = 0.034) and left ventricular myocardium (c; *p* = 0.0072) from mice with MI and controls. Data are shown as box plots; boxes indicate the interquartile range, and horizontal lines indicate the median. Individual points displayed on the box plots represent all measured technical replicate wells (three technical wells per biological sample across 30 human subjects per group and 9 mice per group), whereas statistical *p* values were calculated after averaging technical replicates at the independent biological-sample level.

To explore the bioinformatics findings experimentally, *IDI1*, one of the prioritized computational candidates, was assessed at the expression level in human samples and in a mouse MI model. In human peripheral blood, *IDI1* transcript levels were significantly higher in patients with MI than in healthy controls (*p* = 0.004) (Figure 6a). In mice, *Idi1* expression was also increased in peripheral blood (*p* = 0.034) (Figure 6b) and in infarcted left ventricular myocardium after MI induction (*p* = 0.0072) (Figure 6c).

## 3. Discussion

Epidemiologic studies have established a close association between NAFLD and MI [17,18], consistent with shared metabolic disturbances along the liver–heart axis [9]. However, the molecular mechanisms linking these conditions remain incompletely characterized, and clinically useful transcriptomic biomarkers for their co-occurrence are lacking. We therefore examined shared gene signatures across multiple cohorts, explored computational candidate prioritization across transcriptomic datasets, and evaluated the leading candidate experimentally.

Mechanistically, within the broader context of the liver–spleen and liver–heart axes, the identified five-gene signature (*IDI1*, *SULF2*, *GADD45A, AQP1*, and *LIN7A*) aligns with key pathways driving cIMT progression—a validated surrogate of subclinical atherosclerosis. Specifically, *IDI1*-dependent modulation of the mevalonate pathway may promote smooth-muscle proliferation and cholesterol flux, while *SULF2*-mediated alterations in heparan-sulfate signaling influence endothelial growth-factor responses relevant to intimal expansion. Furthermore, *GADD45A* reflects oxidative and inflammatory stress that accelerates vascular aging, and *AQP1* contributes to endothelial migration and angiogenic remodeling associated with plaque initiation. Although less characterized, *LIN7A* may affect junctional stability and polarity in early endothelial dysfunction. These transcriptomic perturbations are further amplified by chronological aging, which reduces vascular repair capacity and enhances susceptibility to metabolic and inflammatory cues. Consistent with this model, NAFLD patients exhibit increased cIMT across age strata, supporting the concept that hepatic transcriptional dysregulation interacts with age-dependent vascular vulnerability to promote MI risk. Together, these data suggest that the NAFLD–MI gene signature captures a continuum of metabolic–vascular injury that manifests clinically as accelerated cIMT progression in aging individuals.

By combining WGCNA with Mfuzz clustering across ordered cross-sectional groups, we identified 53 genes shared between NAFLD- and MI-related candidate sets. Previous studies have largely characterized NAFLD–MI comorbidity through broad metabolic phenotypes, including insulin resistance, obesity, and chronic inflammation [8]. Our cross-sectional pattern analysis additionally identified systematic expression differences across the ordered healthy, steatosis, and NASH groups. These patterns indicate cross-sectional transcriptional differences across the ordered clinical groups and should not be interpreted as longitudinal progression within individuals. This interpretation is consistent with the view that NAFLD may impose sustained metabolic and inflammatory stress on the cardiovascular system [19,20,21].

Among the five consensus markers (*SULF2*, *GADD45A*, *LIN7A*, *IDI1*, and *AQP1*), *IDI1* showed the strongest experimental support. *IDI1* encodes isopentenyl-diphosphate delta isomerase, an enzyme involved in mevalonate and isoprenoid biosynthesis. The mevalonate pathway contributes to cholesterol synthesis and protein prenylation and may influence hepatic lipid metabolism, vascular smooth-muscle responses, and atherogenesis [22]. We therefore hypothesize that altered *IDI1* expression may link metabolic dysregulation with cardiovascular injury through lipid-related or inflammatory pathways. This mechanistic interpretation remains provisional. Nevertheless, increased *IDI1* expression in peripheral blood from patients with MI and in blood and infarcted myocardium from mice with MI provided experimental support for the transcriptomic association.

The other four signature genes also have plausible links to metabolic or vascular injury. *SULF2* modifies heparan sulfate proteoglycans and can influence growth-factor signaling, including FGF and VEGF pathways, as well as endothelial responses [23,24]. *GADD45A* participates in cellular responses to oxidative stress and DNA damage, processes implicated in atherogenesis [25]. *AQP1* is best known as a water channel but has also been linked to endothelial-cell migration, angiogenesis, and cellular metabolism relevant to NAFLD and cardiovascular disease [26,27]. Evidence directly connecting *LIN7A* with NAFLD and MI is limited. As a scaffold involved in cell polarity and junctional organization, *LIN7A* may represent a less studied component of this comorbidity, but its specific role requires mechanistic investigation [28].

This study extends previous bioinformatics analyses of NAFLD and MI, which identified candidates such as *TLR2*, *TLR4*, and *MMP9* [14], the NCOR1-PPAR-PINK1 axis [15], and a *PDE4D* risk haplotype [16]. Many earlier studies relied on a single dataset or analytical method and lacked experimental validation. Our analysis used six transcriptomic cohorts across NAFLD, MI, and healthy control groups, applied consensus feature selection with LASSO, random forest, and SVM-RFE, and evaluated *IDI1* by qPCR in human samples and a mouse MI model. This combination reduced dependence on any single analytical method and provided biological support for the leading computational candidate.

The five-gene candidate set showed high apparent discrimination under the analyzed grouping, but the estimates require cautious interpretation. GSE49925 contains no-CAD, CAD, old-MI, and acute-MI phenotypes, whereas the original integrated workflow assigned a dataset-level outcome label; the resulting AUCs therefore do not characterize acute-MI diagnostic accuracy. GSE66360 provided secondary evaluation rather than fully independent validation because it contributed to upstream feature discovery. KNN showed favorable observed performance, but formal uncertainty estimates and pairwise ROC comparisons were not performed. Calibration and decision-curve analyses were exploratory and do not establish calibrated clinical risk, an actionable threshold, improved outcomes, or clinical utility. SHAP values indicate contribution to fitted model predictions, not biological importance or mechanism, and single-gene ROC curves indicate case-control discrimination rather than future-risk prediction.

This study has several limitations. First, the NAFLD and MI datasets comprised different participants, and no prospective NAFLD cohort with baseline molecular measurement and incident-MI follow-up was available; thus, the results do not support prospective risk prediction in NAFLD. Second, GSE49925 contains mixed coronary phenotypes but was assigned a dataset-level outcome label in the original integrated workflow, preventing interpretation of the classifier as an acute-MI diagnostic model. Third, GSE66360 contributed to upstream feature discovery, so its later use represents secondary evaluation rather than fully independent external validation and may overestimate performance. No nested cross-validation, feature-selection stability analysis, AUC confidence intervals, formal ROC comparisons, formal calibration statistics, or clinically prespecified DCA threshold was included. Fourth, WGCNA module-trait *p* values were not multiplicity-adjusted, formal module preservation was not tested, and Mfuzz cluster stability across alternative k values was not assessed. ComBat reduces dataset-associated technical variation but cannot fully disentangle cohort origin from biological status, and PCA provides only qualitative visualization. Fifth, after excluding initial specimens with insufficient RNA quality, qPCR assessed *IDI1* expression after established MI using 30 MI and 30 healthy human samples and 9 analyzed mice per group (without a priori sample-size estimation), with three technical wells averaged per biological sample for statistical comparison. Incomplete clinical covariates, sampling-time information, and the absence of NAFLD-by-MI four-group designs limit disease-specific interpretation. The conventional LAD model had no NAFLD phenotype or *IDI1* perturbation, and therefore supports only an association with acute ischemic injury. Larger prospective cohorts, fully independent datasets, longitudinal sampling, and functional studies are required. The present study does not establish a longitudinal NAFLD–cIMT–MI relationship or a causal role of the five genes in vascular disease.

## 4. Materials and Methods

### 4.1. Data Retrieval and Preprocessing

Microarray gene-expression profiles and corresponding clinical metadata for NAFLD and MI were obtained from the Gene Expression Omnibus (GEO) database [29]. The NAFLD datasets were GSE48452 [30] and GSE89632 [31], and the MI datasets were GSE24591, GSE49925, and GSE66360 [32]. Healthy peripheral-blood expression profiles were obtained from GSE30101 and restricted to saline-control samples. Detailed clinical and technical characteristics of all six GEO cohorts, including platform annotations and mapped features, are summarized in Supplementary Table S1. Gene-expression data were downloaded using the GEOquery package [33]. Probe identifiers were mapped to official gene symbols using the corresponding GEO Platform (GPL) annotation files. When multiple probes mapped to the same gene, their expression values were averaged. Samples were categorized according to the available clinical annotations: Healthy, Steatosis, and NASH for NAFLD; Control and AMI for MI; and healthy saline controls for GSE30101. For analyses requiring cross-cohort integration, datasets were restricted to shared genes before batch correction. In the model-development stage, GSE24591, GSE30101, and GSE49925 were pooled, and cross-platform batch effects were corrected using the ComBat algorithm in the sva package [34,35], with disease status retained as a biological covariate. GSE66360 was not included in this model-development integration and was processed separately for subsequent secondary evaluation. Principal component analysis (PCA), implemented in FactoMineR, was used to assess batch-effect correction visually.

### 4.2. Differential Expression Analysis

Differential expression analysis was performed using the linear-modeling framework in the limma package [36]. NAFLD samples were classified as healthy, steatosis, or non-alcoholic steatohepatitis (NASH) and compared pairwise using an intercept-free design matrix. For MI, differentially expressed genes (DEGs) between control and MI samples were identified in GSE66360. Linear models were fitted using lmFit and moderated using empirical Bayes estimation with eBayes. DEGs were defined by |log_2_ fold change| >1 and an adjusted *p* value (false discovery rate [FDR]) <0.05; *p* values were adjusted using the Benjamini–Hochberg procedure. Volcano plots generated with ggplot2 were used to display DEG distributions and expression patterns.

### 4.3. Weighted Gene Co-Expression Network Analysis (WGCNA)

Unsigned weighted co-expression networks were constructed using the WGCNA package to identify disease-associated gene modules [37]. Genes were ranked by expression variance; the top 50% most variable genes were retained for the two NAFLD cohorts, whereas the top 25% most variable genes were retained for the two MI cohorts. Potential outlier samples were identified by hierarchical clustering. The soft-thresholding power was selected using pickSoftThreshold, and topological overlap matrices were calculated with blockwise modules using a minimum module size of 30. Pearson correlations between module eigengenes and numerically coded clinical traits were calculated with nominal *p* values and displayed as annotated heatmaps. These correlations were used for exploratory module selection; *p* values were not adjusted for the number of module-trait tests, and formal module-preservation analysis across cohorts was not performed.

### 4.4. Cross-Sectional Gene Expression Pattern Clustering and Functional Enrichment Analysis

Expression patterns were evaluated across the ordered cross-sectional healthy, steatosis, and NASH groups. Candidate genes were defined as the union of genes in key WGCNA modules and DEGs. For each disease stage, expression matrices contained the mean expression value of each gene. Expression patterns were grouped using the fuzzy c-means soft-clustering algorithm in the Mfuzz package [38], and the elbow method was used to set k = 6. Gene clusters with selected expression patterns were retained for downstream analysis. Gene ontology (GO) enrichment analysis (ontology = “ALL,” *p* < 0.05, q < 0.05) was performed with clusterProfiler [39] after gene symbols were converted to ENTREZ identifiers using org.Hs.eg.db. Genes from selected expression clusters in the two NAFLD cohorts were advanced to cross-disease integration.

### 4.5. Identification of Cross-Disease Consensus Candidate Genes

To identify molecular features shared by MI and NAFLD, candidate gene sets were defined for each condition and then intersected. The MI gene set was obtained by intersecting genes from WGCNA modules associated with MI (the brown module in GSE66360 and the green module in GSE24591) with upregulated DEGs in GSE66360 (adjusted *p* < 0.05 and logFC >0). Thus, GSE66360 contributed to upstream MI candidate-gene discovery. The NAFLD gene set comprised genes from selected expression clusters identified independently in the two NAFLD cohorts: clusters 2, 3, and 5 in GSE48452 and clusters 2, 4, 5, and 6 in GSE89632, followed by gene-level integration across cohorts. The MI and NAFLD candidate gene sets were intersected using the VennDetail package to identify shared cross-disease candidates for subsequent feature selection and modeling.

### 4.6. Feature Selection, Exploratory Classification, and Performance Evaluation

Three machine learning algorithms were applied to the candidate genes to identify consensus computational features: (1) least absolute shrinkage and selection operator (LASSO) regression with cross validation, selecting nonzero coefficients at lambda.min [40]; (2) random forest (RF; 500 trees), with features ranked by mean decrease in Gini impurity; and (3) support vector machine-recursive feature elimination (SVM-RFE) with 5-fold cross-validation. The consensus signature was defined as the intersection of features selected by all three methods, using the top 10 RF-ranked genes, and was visualized with the VennDetail package.

Eight machine learning classifiers (logistic regression [LR], decision tree [DT], RF, K-nearest neighbor [KNN], support vector machine [SVM], extreme gradient boosting [XGBoost], Light Gradient Boosting Machine [LightGBM], and neural network [NNET]) were developed using the consensus signature. After batch-effect correction, samples from GSE24591, GSE30101, and GSE49925 were pooled and stratified by outcome, then randomly partitioned into a training set (70%) and an internal test set (30%). GSE66360 was excluded from model fitting and internal data partitioning and was subsequently used as a secondary evaluation cohort. Because GSE66360 had contributed to upstream candidate-gene discovery, its use at this stage did not constitute fully independent external validation. Model performance was assessed using confusion matrices, receiver operating characteristic (ROC) curves and the corresponding area under the curve (AUC), calibration curves, and decision curve analysis (DCA). SHapley Additive exPlanations (SHAP) analysis quantified the contribution of each feature to model predictions, and single-gene ROC analyses assessed case-control discrimination of individual candidate genes.

### 4.7. Establishment of a Myocardial Infarction Animal Model

MI was induced by permanent ligation of the left anterior descending (LAD) coronary artery. Briefly, male C57BL/6 mice (20–25 g) were anesthetized with 2% inhaled isoflurane and mechanically ventilated. The heart was exposed by left lateral thoracotomy through the fourth intercostal space and pericardial incision. The LAD artery was permanently occluded approximately 2 mm below the left atrial appendage using a 6-0 silk suture. Regional myocardial blanching and marked ST-segment elevation on electrocardiography (ECG) confirmed successful ischemia. Sham-operated mice underwent the corresponding control procedure. After surgery, mice recovered on a temperature-controlled heating pad before return to standard specific pathogen-free housing. Mice were euthanized by isoflurane overdose 24 h after surgery in accordance with institutional animal care guidelines. Peripheral blood and left ventricular tissue were collected postmortem for molecular analyses.

### 4.8. Quantitative Real-Time PCR (qPCR) Expression Assessment

From the initially collected clinical specimens (62 human participants) and animal cohorts (13 mice per group), samples failing RNA quality control or lacking complete triplicate Ct measurements were excluded prior to analysis, yielding a final analyzed set of 30 patients with established MI and 30 healthy controls (60 human subjects in total) and 9 mice per group. While all triplicate technical-well measurements (90 wells per human group and 27 wells per mouse group) are plotted in Figure 6 to display raw assay distribution, the three technical wells were averaged for each biological sample prior to statistical testing. Because both the clinical and animal experiments were designed as exploratory expression assessments, no formal a priori statistical power or sample-size calculation was performed, and sample sizes were determined by sample availability. For each biological sample, three technical qPCR wells were averaged before statistical analysis. RNA was reverse-transcribed, and SYBR Green qPCR was performed with GAPDH as the endogenous control (Thermo fisher, Waltham, MA, USA), with amplification specificity verified by melting-curve analysis (Supplementary Figure S2). ΔCt was calculated as Ct(target)—Ct(GAPDH), and relative expression was calculated as 2^^−ΔCt^ [41]; no separate calibrator or ΔΔCt calculation was used. Primer sequences were: mouse GAPDH (F: TGTGTCCGTCGTGGATCTGA, R: TTGCTGTTGAAGTCGCAGGAG); mouse IDI1 (F: CCTGTTACAGCAGAGATCAG, R: GCTTCACACCAATGGCGTT); human GAPDH (F: GTGGACCTGACCTGCCGTCTAG, R: GAGTGGGTGTCGCTGTTGAAGTC); and human IDI1 (F: GCCGAGCTTGAGGAAAGTGACG, R: TCTGGAGGAACCTCTTCCAAGGG). The human comparison was an exploratory expression assessment after established MI and was not a prospective biomarker-validation study.

### 4.9. Statistical Analysis

All computational and statistical analyses were performed in R version 4.4.2. The eight machine learning models were implemented with the relevant R packages, and bioinformatics analyses used limma, WGCNA, Mfuzz, clusterProfiler, glmnet, sigFeature, pROC, rmda, VennDetail, and FactoMineR. For high-dimensional analyses, including differential expression and functional enrichment, multiple comparisons were adjusted using the Benjamini–Hochberg false discovery rate procedure. For experimental data, continuous variables were assessed for normality and homogeneity of variance. Two independent groups were compared using Student’s t test or the Mann–Whitney U test, as appropriate. Multiple groups were compared using one-way analysis of variance or the Kruskal-Wallis test with appropriate post hoc adjustment. All tests were two-sided, and *p* < 0.05 was considered statistically significant.

## 5. Conclusions

This study identified shared disease-associated transcriptomic features between independent NAFLD and MI cohorts and derived five consensus computational candidates for exploratory classification. IDI1 expression was higher in samples from established human MI and acute mouse ischemic injury, providing expression-level, hypothesis-generating evidence. The findings do not establish prospective MI risk prediction in NAFLD, acute-MI diagnostic accuracy, clinical utility, specificity to NAFLD-associated MI, or a causal role for IDI1.

## Data Availability

The datasets analyzed in this study are available in the GEO repository under accession numbers GSE48452, GSE89632, GSE24591, GSE49925, GSE66360, and GSE30101. Experimental data are available from the corresponding author upon reasonable request.

## Supplementary Materials

The following supporting information can be downloaded at: https://www.mdpi.com/article/10.3390/ijms27209390/s1.

## Abbreviations

The following abbreviations are used in this manuscript:

AMI	acute myocardial infarction
ASCVD	atherosclerotic cardiovascular disease
AUC	area under the curve
BH	Benjamini–Hochberg
ComBat	empirical Bayes batch-correction method
Ct	cycle threshold
CVD	cardiovascular disease
DCA	decision curve analysis
DEG	differentially expressed gene
DT	decision tree
FDR	false discovery rate
FCM	fuzzy C-means
FIB-4	fibrosis-4 index
FLI	fatty liver index
GAPDH	glyceraldehyde-3-phosphate dehydrogenase
GEO	Gene Expression Omnibus
GO	Gene Ontology
GPL	GEO Platform
GS	gene significance
HR	hazard ratio
IR	insulin resistance
KNN	K-nearest neighbor
LASSO	Least Absolute Shrinkage and Selection Operator
LightGBM	Light Gradient Boosting Machine
limma	Linear Models for Microarray Data
log_2_FC	log_2_ fold change
LR	logistic regression
MASLD	metabolic dysfunction-associated steatotic liver disease
Mfuzz	soft clustering via fuzzy C-means (R package)
MI	myocardial infarction
MM	module membership
NAFLD	non-alcoholic fatty liver disease
NASH	non-alcoholic steatohepatitis
NNET	neural network
OR	odds ratio
PCA	principal component analysis
qPCR	quantitative real-time PCR
RF	random forest
ROC	receiver operating characteristic
SHAP	SHapley Additive exPlanations
SVM	support vector machine
SVM-RFE	support vector machine recursive feature elimination
TOM	topological overlap matrix
VennDetail	R package for Venn/UpSet diagrams
WGCNA	weighted gene co-expression network analysis
XGB	extreme gradient boosting
