# Supplementary material for: Cross-Disease Transcriptomic Signatures Shared by NAFLD and MI: Multicohort Machine-Learning Analysis and Exploratory IDI1 Expression Assessment
Source: Int J Mol Sci. 2026 Oct 21;27(20):9390. doi: 10.3390/ijms27209390 (PMC13704942; doi:10.3390/ijms27209390)
Supplement: Supplementary file 1 [file ijms-27-09390-s001.zip › Supplementary Table S1.pdf]

Supplementary Table S1. Clinical and technical characteristics of the transcriptomic cohorts included in this study

GEO cohort	Samples/profiles used	Age	Sex	Disease/control definition	Disease severity/relevant clinical indices	Tissue/sample type	Platform	Comorbidities	Medication exposure	Other relevant characteristics
GSE24591 / GSE24519	38 expression profiles: 34 AMI profiles derived from 17 patients and 4 control profiles	AMI: individual age available in GEO metadata; controls comprised donors from age strata 28–45, 45–60 and >60 years	AMI: individual sex available in GEO; control donor pools were balanced for male and female subjects across age groups	First acute myocardial infarction (FAMI), with blood collected within 6 h of symptom onset; patients had no previous evidence of coronary artery disease.	Number of diseased vessels, CK peak, troponin I on admission, CRP and IL-6 were available for AMI subjects; no standardized Killip/GRACE score reported	Platelets isolated from peripheral whole blood	GPL2895, GE Healthcare/Amersham Biosciences CodeLink Human Whole Genome Bioarray	Previous cardiovascular disease was excluded; other comorbidities were not systematically reported	NR	The 34 AMI profiles represent duplicate A/B profiles from 17 individuals; therefore, the 38 arrays do not represent 38 independent participants. Control profiles were generated from pooled

				Controls were healthy blood donors without cardiovascular disease						healthy-donor material
GSE30101	208 saline-control expression profiles included in the present analysis*	Healthy adult volunteers; approximately 18–64 years in the study population	Both sexes; aggregate sex distribution for the 208-profile subset was not separately reported	Healthy volunteers receiving placebo (saline) injections; no cardiovascular disease phenotype was used to define this cohort	Not applicable	Peripheral whole blood; repeated longitudinal blood sampling	GPL6947, Illumina HumanHT-12 v3.0 Expression BeadChip	Participants were recruited as healthy adults; detailed subject-level comorbidity information was not	Saline placebo was the study intervention; background medication exposure was NR	This was a longitudinal vaccination/placebo study. Multiple transcriptional profiles were obtained from the same individuals at different time points; consequently,

								reported in GEO		208 profiles do not correspond to 208 independent participants
GSE49925	338 subjects; 60 AMI and 278 non-AMI participants in the original study	62 ± 11 years overall; approximately 26–85 years. AMI: 65 ± 11; non-AMI: 61 ± 11 years	65% male overall; AMI approximately 78% male and non-AMI approximately 62% male	Subjects with suspected or confirmed CAD undergoing cardiac catheterization. AMI was diagnosed according to standard universal criteria. Significant	Quantitative coronary angiography and Gensini score were available. Mean Gensini score: 35 ± 54 overall, 48 ± 54 in AMI and 32 ± 54 in non-AMI subjects. LVEF was	Peripheral whole blood collected in PAXgene tubes immediately before angiography	GPL10558, Illumina HumanHT-12 v4.0 Expression BeadChip	Diabetes 36%; hypertension 77%; dyslipidemia 78%; chronic heart failure 23%; previous MI 32%; current smoking approximately 22%;	Aspirin approximately 83%; beta-blockers 67%; clopidogrel 55%; ACE inhibitor/ARB 61%; statins 78%	Clinical characteristics were extensively characterized in the original publication, but GEO public sample annotations were restricted mainly to sex, ethnicity, and technical variables because of

				CAD was defined as ≥50% stenosis in at least one major epicardial vessel	approximately 54 ± 11% overall			history of CABG approximately 21%		IRB/privacy restrictions. Mean follow-up was approximately 2.4 years
GSE66360	99 profiles: 49 AMI and 50 controls	Published microarray subsets: controls, mean age approximately 28 years; AMI, approximately 59–62 years	Published microarray subsets: controls approximately 41–44% male; AMI approximately 76–91% male	AMI cases satisfied clinical criteria including ischemic symptoms, ST-segment elevation, angiographic evidence of obstructive	No standardized disease-severity score such as Killip, GRACE, or Gensini score was publicly available for the complete GEO cohort	Enriched circulating endothelial cells isolated from peripheral blood	GPL570, Affymetrix Human Genome U133 Plus 2.0 Array	Complete patient-level comorbidity data were not publicly available for all 99 GEO profiles	Complete patient-level medication exposure was not publicly available for all 99 GEO profiles	GEO contains 99 profiles divided into discovery and validation sets. Demographic summaries reported in the original publication pertain to the analyzed discovery/valida

				CAD, and positive cardiac biomarkers. Controls were healthy volunteers						tion microarray subsets and do not provide complete individual-level clinical information for all 99 GEO samples

*For GSE30101, the GEO SuperSeries contains a larger longitudinal vaccine/placebo study; only the 208 saline-control profiles selected for the present analysis are summarized here.

Abbreviations: ACEI, angiotensin-converting enzyme inhibitor; AMI, acute myocardial infarction; ARB, angiotensin receptor blocker; BMI, body mass index; CABG, coronary artery bypass grafting; CAD, coronary artery disease; CK, creatine kinase; CRP, C-reactive protein; FAMI, first acute myocardial infarction; GEO, Gene Expression Omnibus; GRACE, Global Registry of Acute Coronary Events; IL-6, interleukin-6; LVEF, left ventricular ejection fraction; NR, not reported or not publicly available.
